# *De novo* microduplication of *CHL1* in a patient with non-syndromic developmental phenotypes

**DOI:** 10.1186/s13039-015-0170-3

**Published:** 2015-08-16

**Authors:** Orazio Palumbo, Rita Fischetto, Pietro Palumbo, Francesco Nicastro, Francesco Papadia, Leopoldo Zelante, Massimo Carella

**Affiliations:** Laboratorio di Genetica Medica, IRCCS Casa Sollievo della Sofferenza, San Giovanni Rotondo, (FG) Italy; Unità Operativa Malattie Metaboliche Genetica Medica, P.O. Giovanni XXIII, A.O.U. Policlinico Consorziale, Bari, Italy

## Abstract

**Background:**

The *CHL1* gene codes for a member of the L1 family of neural cell adhesion molecules. It is highly expressed in the central and peripheral nervous system playing an important role in the building and functioning on the brain. CHL1 proteins are also involved in axonal migration, synaptic formation and plasticity. In mice, functional studies showed that the haploinsufficiency of Chl1 gene in the developing brain results in cognitive deficits suggesting that the *CHL1* gene at 3p26.3 is a candidate for an autosomal form of intellectual disability. Furthermore, in humans deletions of *CHL1* have been described in patients with neurodevelopmental delay characterized by learning and language difficulties, seizures. Less is known about the potential effect of *CHL1* overexpression, and microduplications of *CHL1* have been rarely identified.

**Case presentation:**

In this report, we describe a male patient with a phenotype characterized by developmental delay, symptoms of hyperactivity, short attention span and speech delay. In addition, minor facial dysmorphic features have been observed. Chromosomal microarray analysis revealed a rare *de novo* 0.85 Mb microduplication on the short arm (p26.3) of chromosome 3, encompassing a single gene, *CHL1*. To the best of our knowledge, duplication of chromosome 3p26.3, including only the *CHL1* gene, has been described in only one intellectually disabled girl with epilepsy. The duplication described here is the smallest reported so far. In addition, this is the first report describing a patient in which the *CHL1* duplication is a *de novo* event.

**Conclusions:**

The clinical and molecular findings reported here are useful to provide further evidence that *CHL1* is a dosage sensitive gene suggesting that not only the deletion but also its duplication can cause non-syndromic neurodevelopmental phenotypes.

## Background

Cell adhesion molecules mediate various interactions between cells and also between cells and the extracellular matrix in developing and mature brain. Thus, they are intimately involved in the regulation of brain development and function. The cell adhesion molecule L1-like (*CHL1*) gene, located at the chromosomal sub-band 3p26.3, codes for a cell adhesion molecule of the immunoglobulin superfamily due to the presence of six Ig-like domains in its extracellular part. *CHL1* is highly expressed in neurons but is also detectable in astrocytes, oligodendrocytes, and Schwann cells [1]. In the developing brain, CHL1 regulates neurite outgrowth [1] and neuronal migration [2], while in mature neurons it accumulates in the axonal membrane and regulates synapse function [3]. Deletions or mutations of *CHL1* have been associated with learning and language difficulties while Chl1+/− and Chl1−/− knockout mouse models have provided evidence that Chl1 may contribute to mental impairment associated with “3p-syndrome” [4, 5]. Less is known about the clinical consequences due to the reciprocal microduplications. Up to date, only one patient, a female, carrier of a microduplication in the *CHL1* gene, inherited from her healthy father, has been reported in literature [6]. The proband showed significant ID, marked speech development delay, generalized tonic-clonic seizures. Being the first patient reported in literature carrier of a duplication encompassing only the *CHL1* gene, and since the duplication was inherited from a healthy parent, it remained unclear whether or not *CHL1* was responsible for the clinical phenotype observed. Here, we report another patient with developmental delay (DD), symptoms of hyperactivity, short attention span and speech delay who has a *de novo* duplicated region, less than 1 megabase in size, encompassing only *CHL1*.

We review the clinical and molecular features of *CHL1* gene duplication cases discussing the function of the gene and its role in the etiology of the observed phenotypes.

## Case presentation

### Case report

The patient is the first child of healthy, non-consanguineous parents. He was born at term after a normal pregnancy by cesarean section. Karyotype was normal male. His younger brothers had normal development and schooling. No family history of congenital anomalies or DD/ID was referred. Development was normal during the neonatal period, no feeding problems were reported. He managed to walk unsupported at the age of 1 year showing, since the first year of life, language problems.

**Fig. 1:**
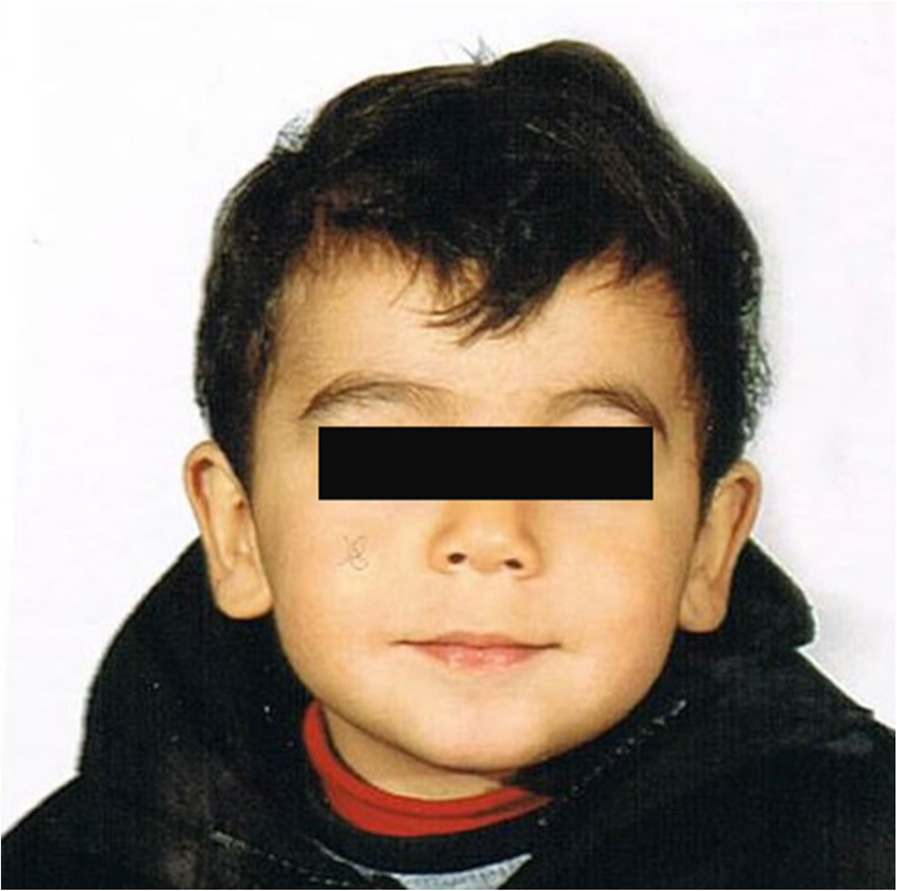
Face of the proband at age of 2 years and 3 months showing mild facial dysmorphic features listed in the text

Medical Geneticist first clinically assessed the patient at 2 years and 3 months. His height was 96 cm (90–97^th^ centile), weight was 15 kg (75–90^th^ centile) and head circumference was 52 cm (50–75^th^ centile). Physical examination revealed minor dysmorphic facial features consisting of mild hypertelorism, down-slanting long palpebral fissures with eversion of lateral third of lower eyelids, long philtrum, thin upper lip, mildly prominent ear lobes (Fig. 1). Sleeping and feeding have been considered normal, as well as brain MRI, EEG, ECG, ocular and audiological assessment, abdomen echography, carpal bone X-rays. Routine blood exams, aminoacidemia/aminoaciduria, urinary organic acids’ panel and acyl-carnitine blood spot were all in normal ranges.

Although the patient’s behavior was friendly and sociable, he showed symptoms of hyperactivity and his attention span was short, but he was too young to confirm a diagnosis of attention deficit hyperactivity disorder (ADHD). Language development was delayed, and he was able to say few words, incorrectly.

At his most recent clinical evaluation, at the age of 3 years and 3 months, the patient had difficulties to focus and sustain his attention. In addition, he shoved hyperactivity and severe speech delay. The family stimulated spontaneous speech associated with few unarticulated words. A neuropediatric examination ruled out a neurological defect. Fragile X screening was unremarkable.

## Results

**Fig. 2:**
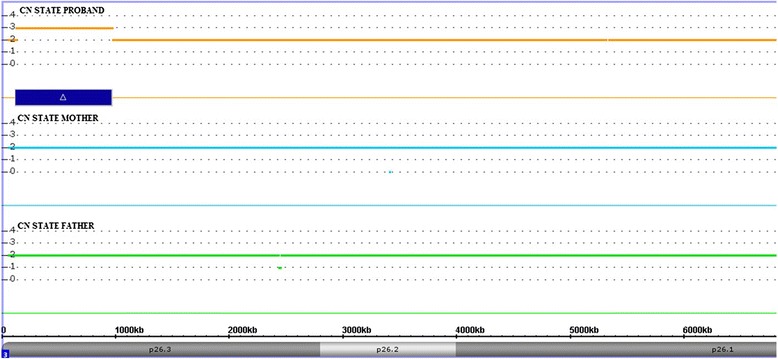
Chromosome microarray analysis performed with the Affymetrix CytoScan HD array and visualized using the Affymetrix Chromosome Analysis Suite version 3.0. Copy number state of each probe is drawn along chromosome 3 from 1 to 6.800.000 bp. The upper panel represents the copy number state of the proband, the middle panel the mother and the lower panel the father. Values of Y-axis indicate the inferred copy number according to probe intensities. Blue bar is the duplicated region identified in the patient

Chromosomal microarray analysis (CMA), performed by high resolution SNP-array, showed an approximately 850 kb duplication on the short arm of chromosome 3 spanning 1,005 probes: arr[hg19]3p26.3(125,931–975,649)x3, encompassing a single coding gene, *CHL1*. The adjacent probes of normal copy number are at position 125,742 distally, and 978,510 proximally, for a maximum duplication size of 852 kb. The duplication was not found in the parents and was thus *de novo* (Fig. 2). We carefully evaluated the microarray results and we excluded the presence of other significant genomic imbalance. The duplication was confirmed by an independent array (data not showed).

## Discussion

In this report, we present the first patient to date reported with a *de novo* whole gene duplication of *CHL1* in 3p26.3 chromosomal region. This is the only duplication encompassing *CHL1* identified in our database of over 3,000 individuals referred for copy number variation (CNV) analysis. In addition, duplications of *CHL1* have not been reported in healthy individuals suggesting a causative role for duplication of this gene in our patient’s abnormal phenotype.

*CHL1* encodes for a 1224-amino acid cell adhesion protein that belongs to the immunoglobulin superfamily due to the presence of six Ig-like domains in its extracellular part. It is highly expressed in neurons but is also detectable in astrocytes, oligodendrocytes, and Schwann cells. Recent work suggests that CHL1 functions as a regulator of synaptic chaperones and vesicle exocytosis. Studies of Chl1-deficient mice have shown impaired sensorimotor gating and neuronal connectivity [7, 8].

In humans, deletions affecting *CHL1* underline a spectrum of neurodevelopmental disorders. To date four familial cases presenting deletions of chromosome 3p26.3 confined to *CHL1* gene have been described. Pohjola and collaborators [9] described a patient showing mild learning problems, microcephaly and growth retardation carrier of a *CHL1* microdeletion inherited from his normal mother; later, a pair of siblings has been reported with features including microcephaly, learning difficulties, and ID who inherited a deletion encompassing only the *CHL1* gene from their asymptomatic father [10]. More recently, Tassano et al. [11] described a maternally inherited 0.95 Mb deletion on the 3p26.3, which removed only the *CHL1* gene, in a male with microcephaly, short stature, mild ID, learning and language delay, and strabismus.

Since the shared deleted region between the reported cases encompassed only the *CHL1* gene, and this latter is highly expressed in the brain, the authors proposed that although the deletion may have incomplete penetrance, the haploinsufficiency of the *CHL1* gene could be the main factor contributing to neurodevelopmental delay observed in these patients.

Of note, a balanced translocation [46,Y,t(X;3)(p22.1;p26.3)] with a breakpoint within intron five of the *CHL1* gene has previously been linked to ID, and an animal model showed that Chl1+/− mice have a phenotype spectrum ranging from wild type to behavioral abnormalities [5].

Taken together, these clinical, molecular and functional data corroborate the hypothesis that *CHL1* is the candidate gene for the cognitive impairments in these patients.

Less is known about the potential effect of *CHL1* overexpression, and microduplications of *CHL1* have been rarely identified.

**Fig. 3:**
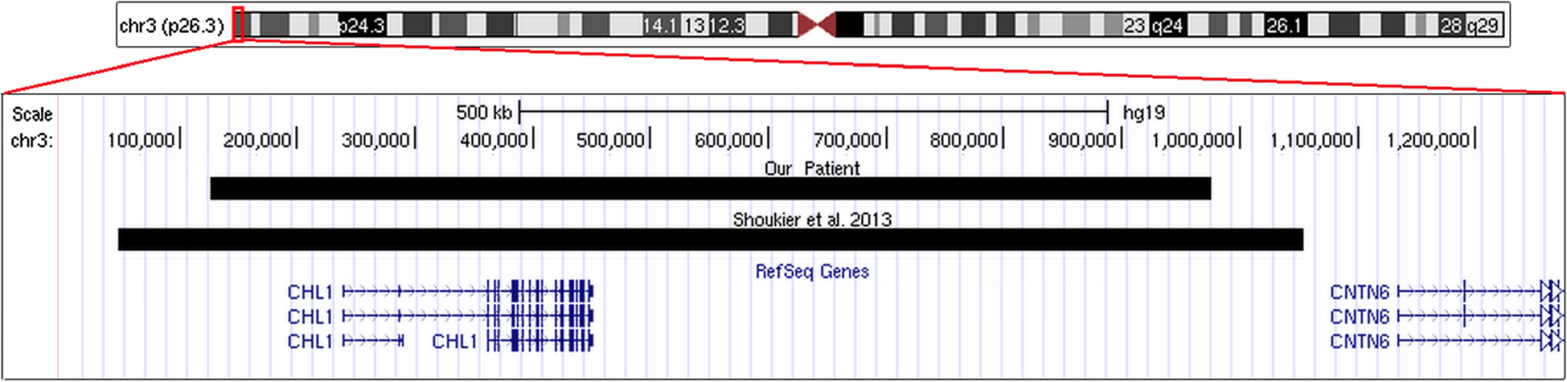
Snapshot of the 3p26.3 region displayed using the UCSC Genome browser [GRCh37/hg19 assembly; http://genome.ucsc.edu] showing the *CHL1* duplications seen in the present patient and in the first patient reported in the literature

Until now, only another patient carrier of a CNV similar for size and chromosomal location to that identified in our patient, has been reported in medical literature by Shoukier et al. [6]. The clinical manifestations of the present case along with the patient reported by Shoukier et al. are listed in Table 1 while the molecular data of the two patients are presented in Fig. 3. A review of clinical features in these two patients revealed overlapping phenotypes, namely, ID/DD and speech delay. Since CHL1 has important regulatory functions both in developing brain and in mature neurons [1–3], we conclude that *CHL1* duplication is likely responsible for the patient’s phenotype. Our observation is also corroborated by the fact that the duplication reported in the present case is the first described to date as *de novo* event in a patient with non-syndromic developmental delay. In addition, copy number variations of genes encoding for neural cell adhesion molecules (NCAM), have been recently published in the literature as responsible for neurodevelopmental disorders further strengthening the hypothesis that chromosomal alteration affecting this family of genes can cause neurodevelopmental disorders [12].

**Table 1:** Summary of the clinical features and molecular data of the reported patients with 3p26.3 microduplication encompassing only the *CHL1* gene

Features	Present case	Shoukier et al. [6]
Dup. size	0.85 Mb	1.0 Mb
Coordinates (hg19)	125,931–975,649	48,914–1,054,209
Inheritance	*de novo*	maternal
Sex and age at diagnosis	M, 2.3 years	F, 16 years
Weight	15 Kg (75–90^th^ centile)	57 Kg (50^th^ centile)
Height	96 cm (90–97^th^ centile)	157 cm (25^th^ centile)
Head circumference	52 cm (50–75^th^ centile)	53.4 cm (25^th^ centile)
DD/ID	+	+
Language delay	+	+
Seizure	-	+
Hyperactivity/attention deficit	+	-
Dysmorphisms	+	-
Delivery	at term	at term
Age at walking	12 months	15 months
Age at first words	20 months	24 months

*Dup* duplication, *M* male, *F* female, + present, − absent, *DD* developmental delay, *ID* intellectual disability, *NR* not reported

Interestingly, epileptic seizures were reported in the patient described by Shoukier et al. [6], whereas symptoms of hyperactivity and short attention span were observed in our. Given the young age of ours patient, we cannot exclude that he will manifest later signs of epilepsy. In addition, since in one of the patients described by Cuoco et al. [10], carrier of a *CHL1* microdeletion, seizure has been observed, we suggest a clinical surveillance for this feature in the patients with CNVs encompassing the *CHL1* gene.

Finally, it is possible that the genes located in the neighboring regions of the duplication have a role in the etiology of the clinical phenotype reported in the patient. In fact, chromosomal rearrangements frequently lead to alteration of the genes’ environment and this may be reflected in a change of expression, referred as a position effect [13]. Of note, located at ~159 kb of the centromeric breakpoint of the 3p26.3 duplication identified in the patient, there is contactin 6 (*CNTN6*) gene (Fig. 2), which could be affected by the rearrangement and related to the phenotype. CNTN6 is a neuronal membrane protein acting as a cell adhesion molecule involved in the formation of axon connection in the developing nervous system. As well as for other members of this protein family, *CNTN6* has been suggested as a disease-causing gene in neurodevelopmental disorders [14]. In mice, *CNTN6* participates in embryonic development and postnatal brain maturation and its deficiency causes profound motor coordination abnormalities and learning difficulties [15]. In humans, microdeletions and microduplications of *CNTN6* have been reported in patients with DD, ID, speech and language delays, atypical autism suggesting that under- and overexpression of this gene is responsible for the observed phenotypes [16]. In agreement with Shoukier et al. [6], we cannot exclude the existence of a position effect on the *CNTN6* gene, as already reported for others copy number variations [17, 18]. Obviously, this observation need to be elucidated by further gene expression studies either on experimental in vivo animal models or on diagnostic material.

## Conclusions

In conclusion, to our knowledge this is the first reported case of an isolated *de novo CHL1* duplication in a patient with a non-syndromic clinical phenotype characterized by developmental and speech delays, hyperactivity and short attention span. Our data are useful to better understand the role that the duplicated gene play in the clinical outcome, corroborating the hypothesis that not only the deletion but also the duplication of *CHL1* is associated with non-syndromic forms of DD/ID.

## Materials and methods

### Snp array analysis

We extracted DNA from the lymphocytes of patient and his parents using BioRobot EZ1 (Qiagen, Solna, Sweden). Genomic screening for copy number variations (CNVs) was carried out using the CytoScan HD array platform (Affymetrix, Santa Clara, CA) as previously described [19]. The array contains more than 2,600,000 CNV markers across the genome, including 750,000 genotype-able single nucleotide polymorphism (SNP) markers. Data analysis were performed using the Chromosome Analysis Suite Software version 3.0: (1) the raw data file (CEL) was normalized using the default options; (2) an unpaired analysis was performed using as baseline 270 HapMap samples in order to obtain Copy numbers value from. CEL files while the amplified and/or deleted regions were detected using a standard Hidden Markov Model (HMM) method. A copy number variation was validated if an abnormal log2 ratio was obtained for at least 25 contiguous probes. DNA sequence information refer to the public UCSC database hg19 assembly (Build GRCh37, February 2009) while molecular karyotype was designated according to ISCN 2013.

### Consent

A copy of the written consent is available for review by the Editor of this journal.
